# Vitamin E analogues as inducers of apoptosis: structure–function relation

**DOI:** 10.1038/sj.bjc.6600981

**Published:** 2003-06-10

**Authors:** M Birringer, J H EyTina, B A Salvatore, J Neuzil

**Affiliations:** 1 German Institute of Human Nutrition, Potsdam-Rehbrücke, Germany; 2 Peptides & Elephants Gmbh, Potsdam-Rehbrücke, Germany; 3 Department of Chemistry and Biochemistry, University of South Carolina, Columbia, SC, USA; 4 Department of Pathology II, Faculty of Health Sciences, University Hospital, Linköping, Sweden; 5 School of Health Sciences, Griffith University, Southport, Queensland, Australia

**Keywords:** vitamin E analogues, apoptosis, synthesis, anticancer effect

## Abstract

Recent results show that *α*-tocopheryl succinate (*α*-TOS) is a proapoptotic agent with antineoplastic activity. As modifications of the vitamin E (VE) molecule may affect its apoptogenic activity, we tested a number of newly synthesised VE analogues using malignant cell lines. Analogues of *α*-TOS with lower number of methyl substitutions on the aromatic ring were less active than *α*-TOS. Replacement of the succinyl group with a maleyl group greatly enhanced the activity, while it was lower for the glutaryl esters. Methylation of the free succinyl carboxyl group on *α*-TOS and *δ*-TOS completely prevented the apoptogenic activity of the parent compounds. Both Trolox and its succinylated derivative were inactive. *α*-tocotrienol (*α*-T3 H) failed to induce apoptosis, while *γ*-T3 H was apoptogenic, and more so when succinylated. Shortening the aliphatic side chain of *γ*-T3 by one isoprenyl unit increased its activity. Neither phytyl nor oleyl succinate caused apoptosis. These findings show that modifications of different functional moieties of the VE molecule can enhance apoptogenic activity. It is hoped that these observations will lead to the synthesis of analogues with even higher apoptogenic and, consequently, antineoplastic efficacy.

Current therapies for neoplastic disease, although often effective in causing remission, frequently lead to deleterious and even life-threatening side-effects. Therefore, there is great interest in developing otherwise nontoxic but effective antineoplastic agents, and inducers of apoptosis may fall into this category of agents (Ferreira *et al*, 2002). In this regard, recent findings suggest that certain analogues of vitamin E (VE), such as *α*-tocopheryl succinate (*α*-TOS), may represent a new class of antineoplastic agents with high selectivity for malignant cells and low toxicity. *α*-Tocopheryl succinate causes apoptotic death of a variety of neoplastic cell lines (Fariss *et al*, 1994; Qian *et al*, 1997; Neuzil *et al*, 1999; Yamamoto *et al*, 2000), whereas neither the redox-active *α*-tocopherol (*α*-TOH) nor its uncharged ester, *α*-tocopheryl acetate (*α*-TOA), is effective (Qian *et al*, 1997; Neuzil *et al*, 2001c). Therefore, the proapoptotic activity appears to be a unique feature of *α*-TOS, and this agent is also effective *in vivo*, inhibiting the growth of colon (Weber *et al*, 2002) and melanoma cancers (Malafa *et al*, 2001), promoting breast cancer dormancy (Malafa and Neitzel, 2000), and suppressing metastasis (Barnett *et al*, 2002). These intriguing observations have prompted us to investigate the molecular properties responsible for this interesting and evidently selective (Neuzil *et al*, 2001b) proapoptotic action of *α*-TOS.

The apparent selective toxicity of *α*-TOS towards malignant *vs* normal cells (Neuzil *et al*, 2001b, c; Weber *et al*, 2002) may be related to its negative charge at neutral pH, a notion supported by findings that its apoptotic action is enhanced at acidic pH (Neuzil *et al*, 2002b). The selective toxicity may thus arise from the fact that pH of the interstitium of most tumours is 6.2–6.5, but in the range of 7.0–7.4 for most normal tissues (Gerweck and Seetharam, 1996). Another possible mechanism by which VE analogues may exert selectivity is because of higher esterase activity of normal compared to malignant cells. Epithelial intestinal cells as well as hepatocytes are known to hydrolyse agents like *α*-TOS, while Jurkat T lymphoma cells are inefficient, whereby accumulating high levels of the toxic agent (Weber *et al*, 2003). The importance of the acidic environment is further supported by a recent observation that chlorambucil, a weak acid, exerted more profound antineoplastic effects when pH of the tumour interstitium was lowered (by hyperglycaemia) in an animal model of breast cancer (Kozin *et al*, 2001).

These findings suggest that modifications of individual functional moieties of VE analogues might modulate proapoptotic activity. We therefore synthesised novel analogues of VE differing from *α*-TOS in the ester side group, in the number and positions of methyl substitutions on the aromatic ring, and in the aliphatic side chain. We show here that these modifications change the original proapoptotic activity of *α*-TOS, either positively or negatively, depending on the type of modifications and their combination.

## MATERIALS AND METHODS

### Chemicals

*α*-D-tocopherol (*α*-TOH; *1*), *γ*-D-tocopherol (*γ*-TOH; *3*), *α*-D-tocopheryl acetate (*α*-TOA; *5*), and *α*-D-tocopheryl succinate (*α*-TOS; *6*) were from Sigma (Castle Hill, NSW, Australia), *α*-trolox (*α*-TroH; *13*) was from Aldrich (Diesenhofen, Germany). *β*-D-tocopherol (*β*-TOH; *2*), *δ*-D-tocopherol (*δ*-TOH; *4*), *α*-tocotrienol (*α*-T3H; *18*) and *γ*-tocotrienol (*γ*-T3H; *19*) were kindly donated by Henkel Inc. (Stockholm, Sweden). Anhydrides of succinic acid, maleic acid, 2-methylsuccinic acid, glutaric acid, 3-methyl-glutaric acid, 3,3-dimethylglutaric acid, and 2,2-dimethylglutaric acid were purchased from Aldrich. Geranylaceton was purchased from Fluka (Diesenhofen, Germany). Methylene chloride was distilled over calcium hydride, under a nitrogen atmosphere. The diazomethane solution in ether was generated from *Diazald*, using a distillation apparatus with Clear-*Seal* glass joints (both items were purchased from Aldrich).

### Synthesis

*General remarks*: Coupled HPLC-electrospray ionisation mass spectrometry (ESI-MS) was performed in negative ionisation. Trimethylsilylether was analysed by GC/MS with electrochemical ionisation. ^1^H NMR and ^13^C NMR spectra were recorded at 300 and 75.47 MHz, respectively, on Bruker AMX 300 with CDCl_3_ as solvent and TMS as internal standard. The carbon ^13^C values for the phytyl side chain of tocopherol derivatives (C1′ to C13′) are not listed, since they are only slightly affected by modifications of the chroman structure and are well established (Rosenau and Kosma, 2001).

*General procedure for tocopheryl- and fatty alcohol carboxylate synthesis*: Appropriate tocopherols/tocotrienols (4 mmol) or fatty alcohols (4 mmol) were dissolved in 15 ml of dry pyridine, and dicarboxylic acid anhydrides were added in a 50% molar excess. The solution was purged with argon and refluxed for 6–12 h depending on the substrates. The reaction mixture was poured into 100 ml of 1 M HCl and extracted three times with diethylether. The combined organic extracts were washed three times with 1 M HCl and dried with Na_2_SO_4_. After solvent evaporation, the crude product was purified by flash chromatography (4 : 2 hexane : ethyl acetate). The yields ranged between 30 and 75%.

*General procedure for methyl ester formation of tocopheryl carboxylates*. The tocopheryl carboxylate (0.16 mmol) and dichloromethane (1 ml) were added to a scratch-free 25 ml Erlenmeyer flask. An ice-cold solution of diazomethane in ether (0.7 ml, 0.3 M) was added drop-wise to the flask with stirring. Nitrogen gas evolved for several minutes, and the solution maintained a yellow colour. The progress of the reaction was monitored by thin layer chromatography; it was generally complete within 5 min. Upon completion, a dilute solution of acetic acid in methanol was added drop-wise, until the yellow colour disappeared and it became colourless. The solvent was removed by rotary evaporation, followed by placement under high vacuum (0.1 Torr). This reaction generally provides quantitative yields of the desired methyl ester (pure by TLC). Flash column chromatography of the product is optional. This can be performed in hexane/ethyl acetate (10 : 1) to provide a sample for analytical characterisation.

#### RRR-α-tocopheryl maleate (α-TOM; 7).

Brown semisolid; ^1^H NMR (CDCl_3_) *δ* 0.84 (d, 3 H), 0.86 (d, *J*=6.6 Hz, 6 H), 0.87 (d, 3 H), 1.25 (s, 3 H), 1.1–1.5 (m, 19 H), 1.5–1.6 (m, 2 H), 1.79 (m, 2 H), 1.98 (s, 3 H), 2.02 (s, 3 H), 2.10 (s, 3 H), 2.60 (t, *J*=6.9 Hz, 2 H), 7.14 (AB, *J*=15.6 Hz, 2 H); ^13^C NMR (CDCl_3_) *δ* 11.8, 12.1, 13.0, 20.6, 24.4, 30.9, 75.2, 117.6, 123.3, 123.9, 126.4, 2 × 135.0, 140.0, 149.8, 2 × 169.2; ESI-MS (*m*/*z*): 527 (MH^+^).

#### RRR-α-tocopheryl 2-methylsuccinate (α-TO2MS; 8).

Yellow semisolid; ^1^H NMR (CDCl_3_) *δ* 0.84 (d, *J*=6.6 Hz, 3 H), 0.86 (d, *J*=6.6 Hz, 6 H), 0.87 (d, *J*=6.4 Hz, 3 H), 1.23 (s, 3 H), 1.1–1.5 (m, 19 H), 1.46 (d, *J*=7.2 Hz, 3 H), 1.5–1.6 (m, 2 H), 1.77 (m, 2 H), 1.96 (s, 3 H), 2.00 (s, 3 H), 2.08 (s, 3 H), 2.60 (t, *J*=6.9 Hz, 2 H), 2.6–2.8 (m, 1 H), 2.9–3.1 (m, 2 H); ^13^C NMR (CDCl_3_) *δ* 11.8, 12.0, 12.9, 17.0, 20.6, 24.4, 31.1, 35.6, 37.0, 75.0, 117.3, 123.2, 124.9, 126.5, 140.4, 149.4, 173.6, 177.7; ESI-MS (*m*/*z*): 543 (MH^+^).

#### RRR-α-tocopheryl glutarate (α-TOG; 9).

Yellow semisolid; ^1^H NMR (CDCl_3_) *δ* 0.84 (d, *J*=6.6 Hz, 3 H), 0.86 (d, *J*=6.6 Hz, 6 H), 0.87 (d, *J*=6.4 Hz, 3 H), 1.23 (s, 3 H), 1.1–1.5 (m, 19 H), 1.5–1.6 (m, 2 H), 1.7–1.9 (m, 2 H), 1.96 (s, 3 H), 2.00 (s, 3 H), 2.09 (s, 3 H), 2.1–2.2 (m, 2 H), 2.54 (t, *J*=7.2 Hz, 2 H), 2.57 (t, *J*=5.1 Hz, 2 H), 2.70 (t, *J*=7.5 Hz, 2 H); ^13^C NMR (CDCl_3_) *δ* 11.8, 12.1, 13.0, 20.2, 20.6, 24.4, 31.1, 32.9, 33.0, 75.2, 117.4, 122.9, 125.1, 126.6, 140.4, 149.4, 171.4; ESI-MS (*m*/*z*): 543 (MH^+^).

#### RRR-α-tocopheryl 3-methylglutarate (α-TO3MG; 10).

Yellow semisolid; ^1^H NMR (CDCl_3_) *δ* 0.84 (d, *J*=6.6 Hz, 3 H), 0.86 (d, *J*=6.6 Hz, 6 H), 0.87 (d, *J*=6.4 Hz, 3 H), 1.23 (s, 3 H), 1.1–1.5 (m, 20 H), 1.17 (d, *J*=6.0 Hz, 3 H), 1.5–1.6 (m, 2 H), 1.7–1.9 (m, 2 H), 1.97 (s, 3 H), 2.01 (s, 3 H), 2.08 (s, 3 H), 2.4 (m, 2 H), 2.58 (t, *J*=7.2 Hz, 2 H), 2.7 (m, 2 H); ^13^C NMR (CDCl_3_) *δ* 11.8, 12.2, 13.0, 19.9, 20.6, 24.4, 27.1, 31.1, 40.2, 40.4, 75.0, 117.4, 123.0, 124.7, 126.6, 140.5, 149.4, 170.8, 178.0; ESI-MS (*m*/*z*): 557 (MH^+^).

#### RRR-α-tocopheryl 3,3-dimethylglutarate (α-TO33DMG; 11)

Yellow semisolid; ^1^H NMR (CDCl_3_) *δ* 0.84 (d, *J*=6.6 Hz, 3 H), 0.86 (d, *J*=6.6 Hz, 6 H), 0.87 (d, *J*=6.4 Hz, 3 H), 1.23 (s, 3 H), 1.1–1.5 (m, 19 H), 1.25 (s, 6 H), 1.5–1.6 (m, 2 H), 1.7–1.9 (m, 2 H), 1.97 (s, 3 H), 2.01 (s, 3 H), 2.08 (s, 3 H), 2.58 (m, 2 H), 2.62 (s, 2 H), 2.79 (s, 2 H); ^13^C NMR (CDCl_3_) *δ* 11.8, 12.3, 13.1, 20.6, 24.4, 28.0, 31.1, 2 × 32.4, 43.9, 44.5, 75.0, 117.3, 123.0, 124.9, 126.7, 140.5, 149.4, 170.8, 177.2; ESI-MS (*m*/*z*): 571 (MH^+^).

#### RRR-α-tocopheryl 2,2-dimethylglutarate (α-TO22DMG; 12)

Yellow semisolid; ^1^H NMR (CDCl_3_) *δ* 0.84 (d, *J*=6.6 Hz, 3 H), 0.86 (d, *J*=6.6 Hz, 6 H), 0.87 (d, *J*=6.4 Hz, 3 H), 1.23 (s, 3 H), 1.1–1.5 (m, 19 H), 1.30 (s, 6 H), 1.5–1.6 (m, 2 H), 1.7–1.9 (m, 2 H), 1.96 (s, 3 H), 2.00 (s, 3 H), 2.08 (s, 3 H), 2.1 (m, 2 H), 2.58 (t, *J*=6.6 Hz, 2 H), 2.65 (m, 2 H); ^13^C NMR (CDCl_3_) *δ* 11.8, 12.1, 13.0, 20.6, 24.4, 24.8, 24.9, 30.0, 31.1, 35.0, 41.6, 75.0, 117.4, 123.0, 124.9, 126.6, 140.4, 149.4, 171.9, 183.1; ESI-MS (*m*/*z*): 571 (MH^+^).

#### Trolox succinate (TroS; 14)

Brown solid; ^1^H NMR (CDCl_3_) *δ* 1.64 (s, 3 H), 1.91 (s, 3 H), 2.00 (s, 3 H), 2.15 (s, 3 H), 2.4 (m, 2 H), 2.6 (m, 2 H), 2.76–3.1 (m, 4 H); ^13^C NMR (CDCl_3_), *δ* 11.3, 12.0, 12.9, 20.5, 25.1, 28.7, 28.9, 31.1, 76.2, 117.2, 123.1, 124.2, 125.4, 140.8, 145.1, 170.3, 172.6, 174.8; ESI-MS (*m*/*z*): 349 (MH^+^).

#### RRR-δ-tocopheryl succinate (δ-TOS; 17)

Yellow semisolid; ^1^H NMR (CDCl_3_) *δ* 0.84 (d, *J*=6.6 Hz, 3 H), 0.86 (d, *J*=6.6 Hz, 6 H), 0.87 (d, *J*=6.4 Hz, 3 H), 1.23 (s, 3 H), 1.1–1.5 (m, 19 H), 1.5–1.6 (m, 2 H), 1.77 (m, 2 H), 2.14 (s, 3 H), 2.60 (t, *J*=6.9 Hz, 2 H), 2.80–2.84 (m, 4 H), 6.6–6.7 (m, 2 H); ^13^C NMR (CDCl_3_) *δ* 16.1, 19.6, 19.7, 30.0, 22.4, 22.6, 22.7, 24.4, 24.8, 28.0, 28.9, 28.9, 31.0, 32.7, 32.8, 37.2, 37.4, 40.2, 75.0, 118.9, 2 × 121, 127.3, 142.3, 149.8, 171.3, 177.8; ESI-MS (*m*/*z*): 501 (MH^+^).

#### R-γ-tocotrienyl succinate (γ-T3S; 20)

Yellow semisolid; ^1^H NMR (CDCl_3_) *δ* 1.26 (s, 3 H), 1.60 (s, 9 H), 1.68 (s, 3 H), 1.5–1.9 (m, 2 H), 1.95–2.2 (m, 12 H), 2.01 (s, 3 H), 2.10 (s, 3 H), 2.71 (m, 2 H), 2.75–2.95 (m, 4 H), 5.11 (m, 3 H), 6.57 (s, 1 H); ^13^C NMR (CDCl_3_) *δ* 11.9, 12.6, 15.9, 16.0, 17.6, 22.2, 2 × 24.1, 25.7, 26.6, 26.8, 28.9, 28.9, 31.0, 2 × 39.7, 40.0, 75.0, 113.3, 118.4, 118.8, 124.2, 124.4, 125.9, 127.1, 131.2, 135.0, 135.2, 141.5, 149.46, 170.5, 171.2; ESI-MS (*m*/*z*): 509 (MH^+^).

#### Phytyl succinate (PYS; 22)

Colourless semisolid; ^1^H NMR (CDCl_3_) *δ* 0.84 (d, *J*=6.6 Hz, 3 H), 0.86 (d, *J*=6.6 Hz, 6 H), 0.87 (d, *J*=6.4 Hz, 3 H), 1.1–1.5 (m, 19 H), 1.70 (s, 3 H), 1.95–2.15 (m, 2 H), 2.6 (m, 4 H), 4.62 (t, *J*=6.9 Hz, 2 H), 5.34 (m, 1 H); ^13^C NMR (CDCl_3_) *δ* 16.3, 2 × 19.7, 2 × 22.7, 24.5, 24.8, 28.0, 28.9, 29.0, 32.6, 32.7, 32.7, 32.8, 36.6, 36.8, 37.3, 37.4, 39.3, 61.8, 117.8, 143.1, 172.3, 178.1; ESI-MS (*m*/*z*): 395 (MH^+^).

#### Oleyl succinate (OS; 23).

Colourless semisolid; ^1^H NMR (CDCl_3_) *δ* 0.88 (t, 3 H), 1.2–1.4 (m, 22 H), 1.62 (m, 2 H), 2.01 (m, 4 H), 2.66 (m, 4 H), 4.09 (t, *J*=6.6 Hz, 2 H), 5.35 (m, 2 H); ^13^C NMR (CDCl_3_) *δ* 14.1, 22.7, 25.8, 2 × 27.2, 28.5,2 × 28.9, 2 × 29.2, 2 × 29.3, 29.4, 29.5, 29.7, 29.8, 31.9, 65.0, 129.8, 130.0, 172.2, 178.1; ESI-MS (*m*/*z*): 367 (MH^+^).

#### RRR-α-tocopheryl succinyl methyl ester (α-TOSM; 24).

Clear colourless oil; ^1^H NMR (CDCl_3_) *δ* 0.85 (d, *J*=6.6 Hz, 3 H), 0.86 (d, *J*=6.3 Hz, 3 H), 0.87 (d, *J*=6.6 Hz, 6 H), 1.24 (s, 3 H), 1.02–1.44, (m, 19 H), 1.46–1.60 (m, 2 H) , 1.74–1.82 (m, 2 H), 1.98 (s, 3 H), 2.02 (s, 3 H), 2.09 (s, 3 H), 2.59 (t, *J*=6.7 Hz, 2 H), 2.77 (m, 2 H), 2.94 (m, 2 H), 3.72 (s, 3 H); ^13^C NMR (CDCl_3_) 11.8, 12.0, 12.9, 19.6, 19.7, 20.6, 21.0, 22.6, 22.7, 24.4, 24.8, 28.0, 28.8, 28.9, 31.1, 32.7, 32.8, 37.3, 37.4, 39.3, 51.9, 75.0, 77.2, 117.3, 123.0, 124.9, 126.6, 140.4, 149.4, 170.9, 172.6.

#### RRR-δ-tocopheryl succinyl methyl ester (δ-TOSM; 25).

Clear colourless oil; ^1^H NMR (CDCl_3_) δ 0.77 (d, *J*=7.1 Hz, 3 H), 0.78 (d, *J*=6.6 Hz, 3 H), 0.80 (d, *J*=6.6 Hz, 6 H), 1.18 (s, 3 H), 0.94–1.39, (m, 19 H), 1.41−1.51 (m, 2 H), 1.62–1.75 (m, 2 H), 2.06 (s, 3 H), 2.63–2.68 (m, 4 H), 2.75-2.80 (m, 2 H), 3.65 (s, 3 H), 6.55 (d, *J*=2.5 Hz, 1 H), 6.60 (d, *J*=2.5 Hz, 1 H); ^13^C NMR (CDCl_3_) *δ* 16.1, 19.6, 19.7, 21.0, 22.4, 22.6, 22.7, 24.2, 24.4, 24.8, 28.0, 29.0, 29.3, 30.9, 32.7, 32.8, 37.3 37.41, 37.43, 39.4, 40.1, 51.9, 76.1, 77.2, 118.9, 120.9, 121.0, 127.3, 142.3, 149.8, 171.6, 172.6.

#### Synthesis of α-2-geranylchromanol. 2, 6, 10-trimethyl-10-hydroxy-2, 6, 11-dodecatriene

Vinyl magnesium bromide (1 M in tetrahydrofuran, 6.4 ml, 6.4 mmol) was added with vigorous stirring under argon at 0–5°C to a solution of geranylaceton (1.2 g, 6.2 mmol) in diethylether (100 ml) over 60 min. The reaction mixture was stirred for additional 30 min and acidified with 1 M HCl to pH 2, and diluted with water to dissolve precipitated salts. This solution was extracted with ether (3 × 100 ml), and the combined ether extracts washed with brine (3 × 50 ml) and dried over Na_2_SO_4_. Ether was removed on a rotavapor to yield yellow oil that was used without further purification.

#### α-2-geranylchromanol (α-T2H; 21)

Vinyl alcohol (0.96 g, 4 mmol) in dioxane (2 ml) was added over 1.5 h at 110°C to a stirred solution of 2,3,5-trimethylhydroquinone (0.42 g, 2.8 mmol) and boron trifluoride etherate (0.7 ml, 5.5 mmol) in dioxane (15 ml) under argon. The reaction mixture was extracted with ethyl acetate (3 × 100 ml). The combined organic layers were washed with water, dried over Na_2_SO_4_, concentrated under vacuum and applied to a silica gel chromatography (hexane : ethyl acetate, 5 : 1) to yield orange oil (285 mg, 30%). ^1^H NMR (CDCl_3_) *δ* 1.16 (s, 3 H), 1.60 (s, 6 H), 2.34 (s, 3 H), 1.95–2.2 (m, 6 H), 1.98 (s, 3 H), 2.01 (s, 3 H), 2.10 (s, 3 H), 2.69 (m, 2 H), 5.05 (m, 3 H); ^13^C-NMR (CDCl_3_) *δ* 11.8, 11.9, 12.0, 17.8, 22.2, 2 × 24.1, 24.2, 25.7, 26.8, 31.0, 39.6, 40.3, 75.1, 118.4, 118.8, 124.4, 124.9, 125.9, 126.9, 131.0, 135.1, 145.2, 149.5; MS (EI) *m*/*z* of trimethylsilylether: 428 (M^+^).

### Cell culture and treatment

The human T lymphoma cell line Jurkat, neuroblastoma cell line HTB11, and the breast carcinoma cell line MCF7 and its caspase-3-expressing variant (Mathiasen *et al*, 1999) were used in the study. Jurkat cells were maintained in the RPMI-1640 medium supplemented with 10% foetal calf serum (FCS) and antibiotics, while the other lines were cultured in DMEM with 10% FCS and antibiotics. Suspension cells (0.5 × 10^6^ ml^−1^) and adherent cells (50–70% confluency) were exposed to individual agents at their concentration of up to 50 *μ*M for varying periods of time and assessed for apoptosis as detailed below.

### Apoptosis evaluation

Apoptosis was routinely assessed by the annexin V-binding method, which is based on affinity of annexin V to phosphatidylserine externalised to the outer leaflet of the plasma membrane early in the course of apoptosis (Neuzil *et al*, 2001a). In brief, suspension cells were harvested by spinning down, while adherent cells were detached by treatment with 2 mM EDTA in PBS and combined with the cells detached during treatment. The cells were then washed with PBS, spun down, resuspended in the binding buffer (10 mM Hepes/NaOH, 140 mM NaCl and 25 mM CaCl_2_, pH 7.4), incubated with 2 *μ*l of annexin V–FITC (PharMingen, San Diego, CA, USA), and analysed by flow cytometry (Becton Dickinson, Rutherford, NJ, USA). In some cases, activation of caspase-3 was assessed as follows. Treated cells were washed, permeabilised with 0.02% saponin in PBS, and incubated with an antibody raised against activated caspase-3 (PharMingen) at room temperature for 60 min. The cells were then washed and incubated with secondary IgG conjugated to FITC at room temperature for additional 60 min, washed, and scored for FITC binding in a flow cytometer. The percentage of apoptotic cells or cells with activated caspase-3 was estimated by gating on the population with high fluorescence.

Unless specified otherwise, the data shown are mean values ±s.d. (*n*=3). The asterisk at individual values indicates statistically significant difference from the control with *P*<0.05, as determined by the Student's *t*-test.

## RESULTS AND DISCUSSION

**Figure 1:**
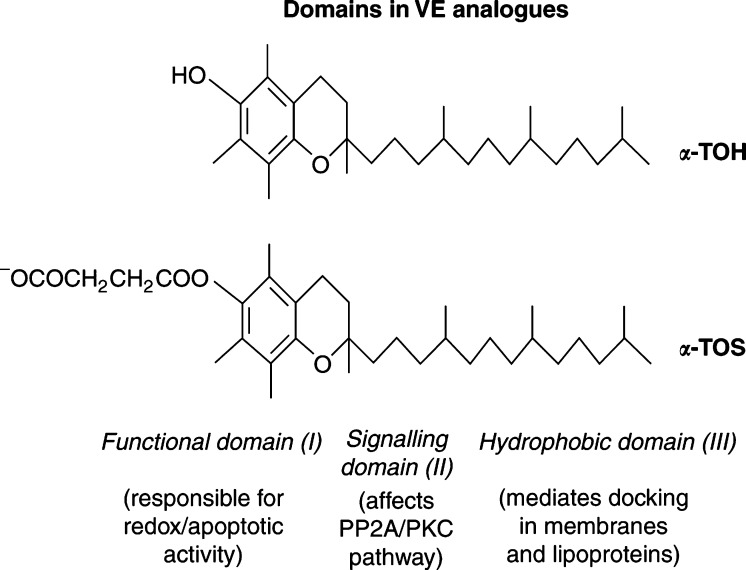
Functional domains in the molecule of VE. Vitamin E analogues (represented here by *α*-TOH and *α*-TOS) comprise three distinct domains, each responsible for a separate ‘function’ of the agent. Domain I, the *functional* domain, decides whether the compound is redox-active or redox-inactive. Domains II, the *signalling* domain, is responsible for effects of the analogues such as deregulation of the protein kinase C/protein phosphatase 2A pathway. Domain III, the *hydrophobic* domain, is responsible for docking of VE analogues in biological membranes and lipoproteins.

**Figure 2:**
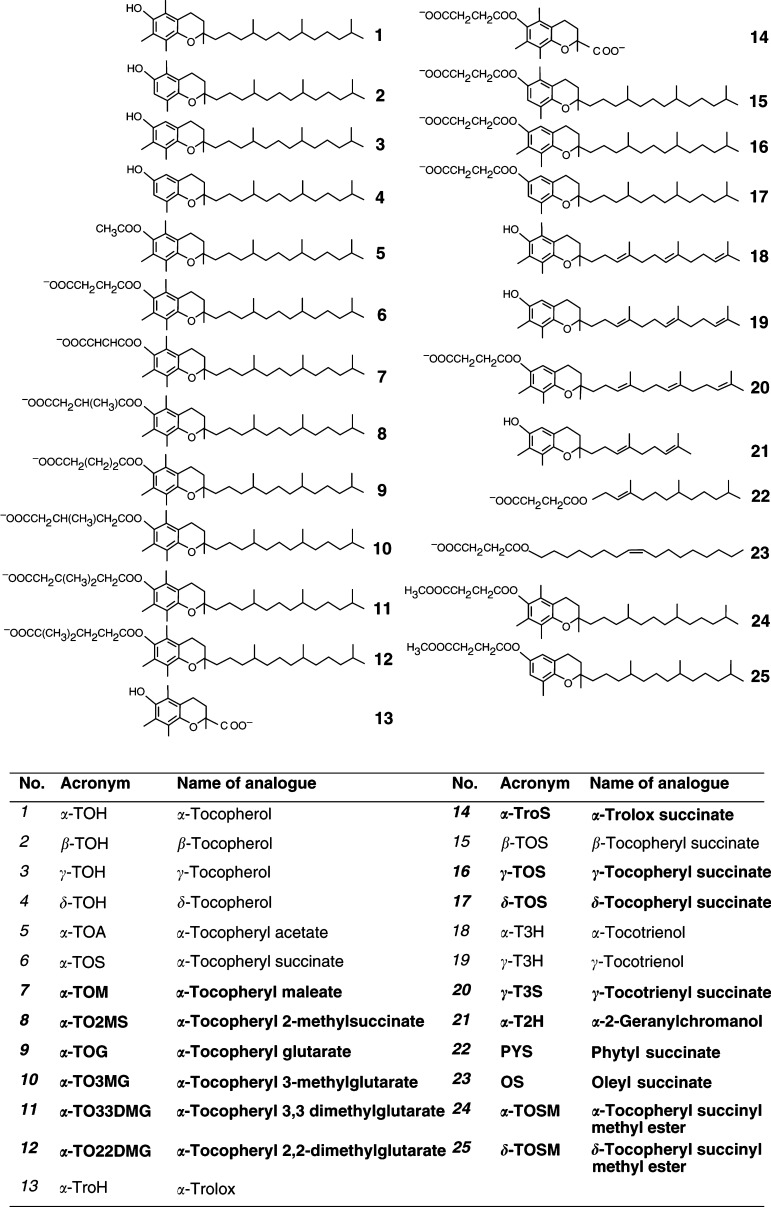
Analogues of VE used in this study. The items shown in bold indicate newly synthesised compounds.

The molecule of VE can be divided into three functionally distinct domains (Figure 1

) (Neuzil *et al*, 2002a). Domain I (the *functional* domain) is essential for the redox activity of VE analogues, which involves the hydroxyl group in position C6 of the chromanol ring structure. Interestingly, *α*-TOH does not induce apoptosis (Qian *et al*, 1997; Neuzil *et al*, 2001c), nor does it when acetylated at C6 (*α*-TOA) (Neuzil *et al*, 2001a). However, succinylation in this position makes *α*-TOH a strong apoptogen (Neuzil *et al*, 2001c). Conversion of *α*-TOH into a charged species may thus play a role in apoptosis induction (Neuzil *et al*, 2002b). We therefore investigated the effects of substituting *α*-TOS with other dicarboxylic acids in this position. Of these analogues, *α*-TOM was more apoptogenic compared to *α*-TOS, whereas the other esters tested were less effective than *α*-TOS in the order *α*-TO2MS >*α*-TOG >*α*-TO3MG >*α*-TO33DMG=*α*-TO22DMG (Figures 2

and 3

**Figure 3:**
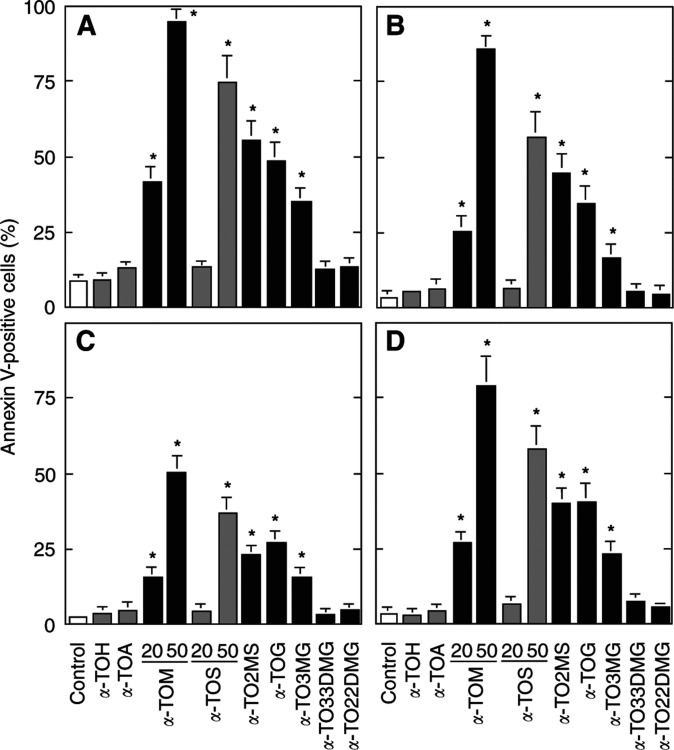
Effect of modifications in domain I of the VE molecule on their apoptogenic activity. Jurkat (**A**), HBT11 (**B**), MCF7 (**C**), and MCF7-C3 cells (**D**) were exposed for 24 h to *α*-TOH, *α*-TOA, *α*-TOM, *α*-TOS, *α*-TO2MS, *α*-TOG, *α*-TO3MG, *α*-TO33DMG, or *α*-TO22DMG (11), and assessed for apoptosis using the annexin V–FITC method.

).

**Figure 4:**
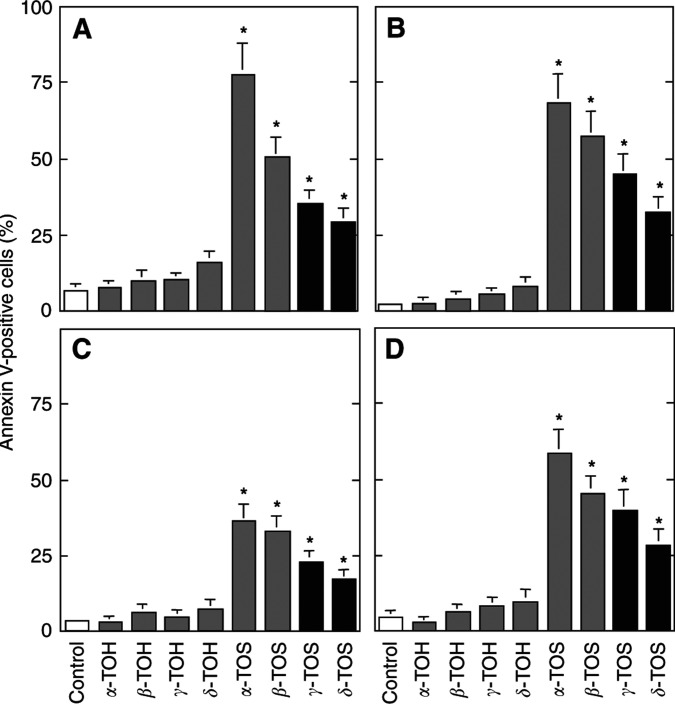
Effect of modifications in domain II of the VE molecule on their apoptogenic activity. Jurkat (**A**), HBT11 (**B**), MCF7 (**C**) and MCF7-C3 cells (**D**) were exposed for 24 h to *α*-TOH, *β*-TOH, *γ*-TOH, *δ*-TOH, *α*-TOS, *β*-TOS, *γ*-TOS, or *δ*-TOS, and assessed for apoptosis using the annexin V–FITC method.

Naturally occurring derivatives of *α*-TOH differ in the number and position of methyl substitutions on the aromatic ring, that is domain II (*signalling* domain). These include *β*-, *γ*- and *δ*-TOH. All of these agents were largely nonapoptogenic (Neuzil *et al*, 2002a) (Figure 4

). Succinylation made them proapoptotic, although the activity of *β*-, *γ*- and *δ*-TOS was lower than that of *α*-TOS, with *δ*-TOS being least apoptogenic (Figure 4). Their efficacy in inhibiting protein kinase C also differs, and is not dependent on their antioxidant capacity (Ricciarelli *et al*, 2001). In addition, the substitution pattern is responsible for the rate of side chain degradation, as *γ*- and *δ*-TOH are degraded much faster than *α*- or *β*-TOH in cell culture (Birringer *et al*, 2001).

**Figure 5:**
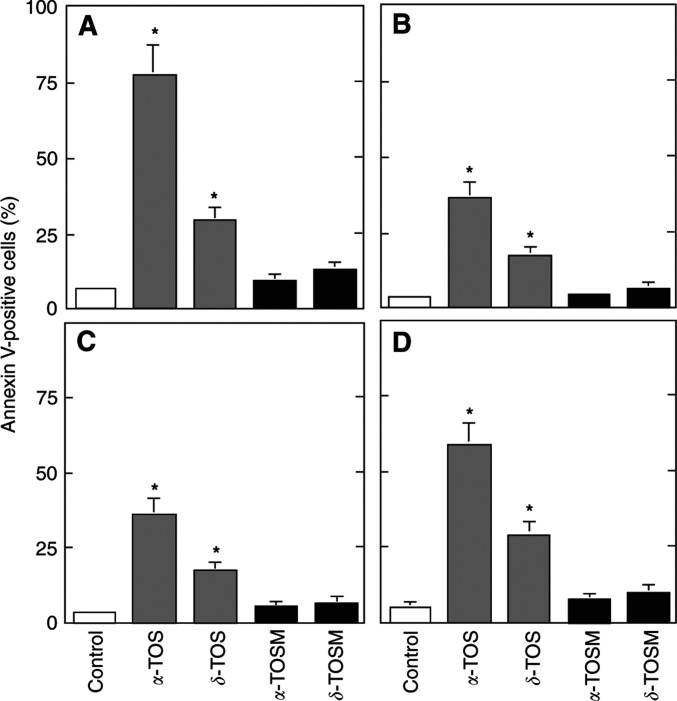
Effect of methylation of the succinyl moiety of VE succinyl analogues on their apoptogenic activity. Jurkat (**A**), HBT11 (**B**), MCF7 (**C**) and MCF7-C3 cells (**D**) were exposed for 24 h to *α*-TOS, *δ*-TOS, *α*-TOSM or *δ*-TOSM, and assessed for apoptosis using the annexin V–FITC method.

To address the importance of the free carboxylic group for apoptogenic activity of VE dicarboxylic acid esters with saturated isoprenyl side chains, we esterified *α*-TOS and *δ*-TOS on the succinyl moiety. The resulting methyl esters were completely inactive as inducers of apoptosis in all cell lines tested (Figure 5

).

**Figure 6:**
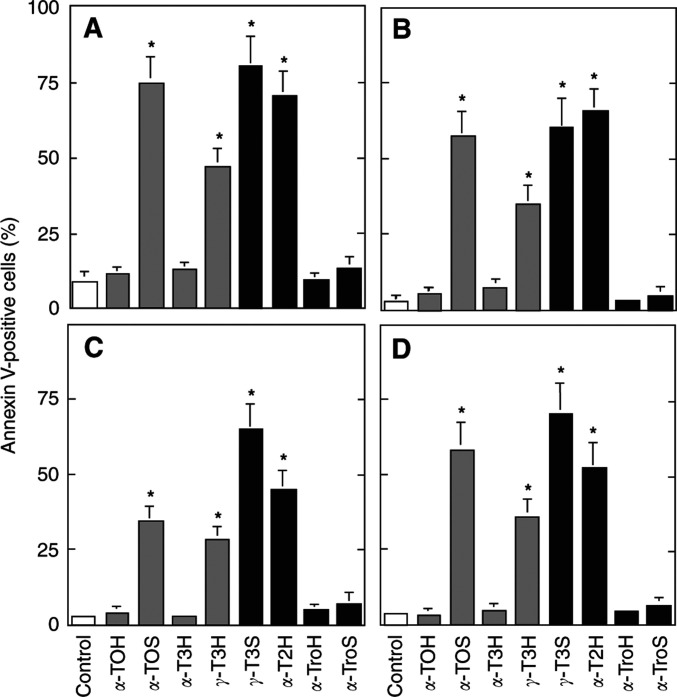
Effect of modifications in domain III of the VE molecule on their apoptogenic activity. Jurkat (**A**), HBT11 (**B**), MCF7 (**C**) and MCF7-C3 cells (**D**) were exposed for 24 h to *α*-TOH, *α*-TOS, *α*-T3H, *γ*-T3H, *γ*-T3S, *α*-T2H, *α*-TroH, or *α*-TroS, and assessed for apoptosis using the annexin V–FITC method.

Several modifications of the aliphatic side chain (domain III, the *hydrophobic* domain) are possible. Its desaturation, in the case of *α*-TOH, gives the naturally occurring *α*-T3H, which is nonapoptotic (Yu *et al*, 1999). *γ*-T3H, however, has a strong apoptogenic activity (Yu *et al*, 1999), which is further enhanced by its conversion to *γ*-T3S (Figure 6

). Interestingly, a derivative of *γ*-T3H with the aliphatic side chain shorter by one isoprenyl unit, *α*-T2H, exerted higher proapoptotic activity than did *γ*-T3H itself (Figure 6). Analogues of VE lacking the aliphatic side chain, that is, *α*-TroH and *α*-TroS did not induce apoptosis, regardless of the succinylation status (Figure 6).

**Figure 7:**
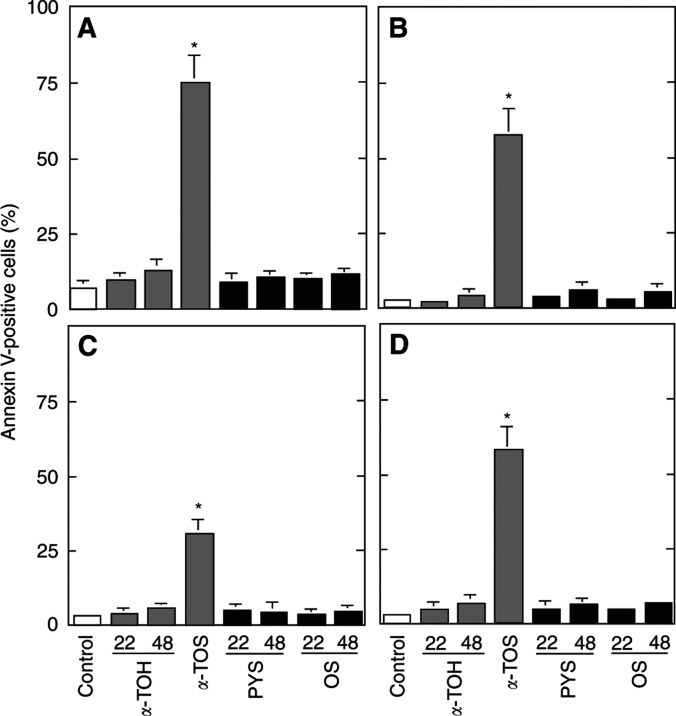
Proapoptotic activity of other structural analogues of VE. Jurkat (**A**), HBT11 (**B**), MCF7 (**C**), and MCF7-C3 cells (**D**) were exposed for 24 h to *α*-TOS, and for 22 or 48 h to *α*-TOH, PYS, or OS, and assessed for apoptosis using the annexin V–FITC method.

Finally, we synthesised succinyl esters of long-chain fatty acids, that is, PYS and OS. Neither of them caused any signs of apoptosis at up to 100 *μ*M and at times of up to 48 h (Figure 7

). These results indicate that presence of an aliphatic group at one end and a chargeable moiety at the other end is not sufficient for apoptosis induction and that presence of the chromanol structure may be essential.

**Figure 8:**
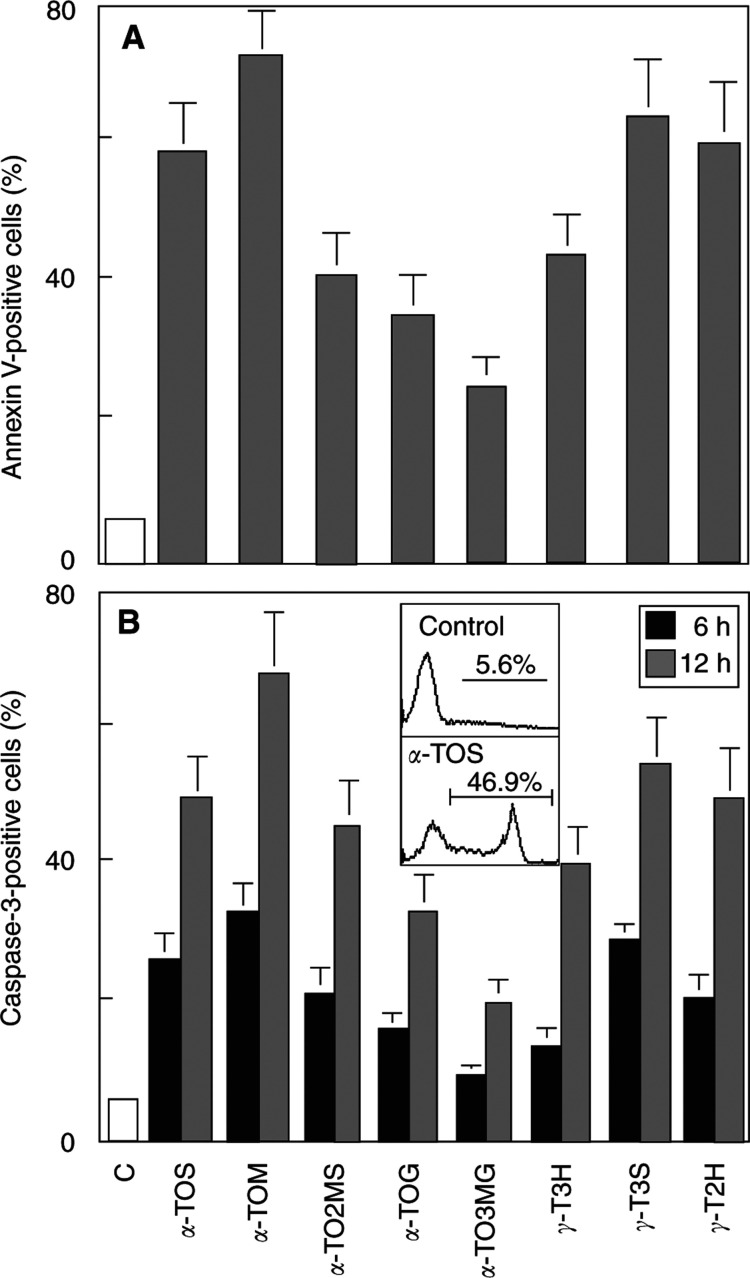
Vitamin E analogues induce apoptosis and activate caspase-3. Jurkat cells were exposed for the time shown to 50 *μ*M each of *α*-TOS, *α*-TOM, *α*-TO2MS, *α*-TOG, *α*-TO3MG, *γ*-T3H, *γ*-T3S, or *γ*-T2H, and assessed for apoptosis extent (**A**) and caspase-3 activation using the antibody specific for activated caspase-3 and a secondary, FITC-conjugated antibody (**B**). The inset in (**B**) shows a typical histogram of flow cytometric evaluation of control cell and cells treated with 50 *μ*M *α*-TOS for 12 h.

Toxicity of *α*-TOS towards cancer cells is known to be governed by apoptotic signalling. To find out whether this form of cell death is also involved in killing by the newly synthesised VE analogues, we exposed Jurkat cells to the agents for increasing time, and analysed the cells for caspase-3 activation, a hallmark of apoptotic signalling. As demonstrated in Figure 8

, all new VE analogues, which were shown above to cause annexin V binding in several cancer cell lines, caused time-dependent activation of caspase-3. Assessment of annexin V binding was carried out on adherent and detached cells pooled together (see Materials and Methods section). It cannot be excluded that some of the cells died by anoikis. However, similar effects wre observed with suspension, Jurkat cells. This strongly suggests that apoptotic cell death is the major mechanism by which these novel agents kill cancer cells.

The data presented herein suggest the following conclusions: (i) Forms of VE with saturated aliphatic chains need to be esterified with a dicarboxylic acid to exert apoptogenic activity. (ii) The extent of proapoptotic effect is greatly dependent on the un-saturation and length of the ester group substituent. Thus, the C6 maleyl-substituted analogue showed the highest activity, which was virtually nondetectable in case of the 2,2-dimethyl- and 3,3-dimethylglutaryl substituents. This may suggest that the apparently less water-soluble agents are less bioavailable for the malignant cells tested. Moreover, *α*-TOM may have higher *in vivo* effectiveness as it is ∼10–20-fold more efficient *in vitro* than the relatively well-studied *α*-TOS. The latter agent exerts antitumour effects against experimental colon cancer at the pharmacologically relevant blood levels of ∼40–50 *μ*M (Weber *et al*, 2002). We would thus expect *α*-TOM to be equipotent with *α*-TOS *in vivo* at its plasma levels of ∼5 *μ*M, that is, similar to those of circulating VE. (iii) Finally, and perhaps most importantly, the apoptogenic activity of *α*-TOS and related derivatives does require a chargeable substituent in position C6. This conclusion is supported by the fact that *α*-TOS and *α*-TOA, while taken up at comparable rates (Cheeseman *et al*, 1998), show a completely different apoptogenic potential (cf Figure 3), and, in particular, by the finding that esterification of the free carboxyl group on the succinyl moiety completely obliterates apoptogenic activity of the parent compounds (cf Figure 5).

One possible mechanism underlying the proapoptotic effects of *α*-TOS and similar compounds may involve membrane destabilisation. *α*-Tocopheryl succinate has an aliphatic side chain, which docks it in the lipid phase (Pussinen *et al*, 2000), and a hydrophilic head group. In support of this notion, we observed detergent-like effects of *α*-TOS using isolated erythrocytes or lysosomes (Neuzil *et al*, 2002b). This suggested that many molecules with an aliphatic chain on one end and a hydrophilic group on the other end could induce apoptosis. To test this, we synthesised succinylated long-chain aliphatic acids, that is, PYS and OS. However, these compounds were nontoxic to all cell lines tested even following prolonged exposure (cf Figure 7). Thus, it appears that the apoptosis-inducing activity may require the presence of the (bulky) chromanol structure, and this may still act through promoting the destabilisation of phospholipid membranes. This idea appears consistent with the finding that *β*-, *γ*- and, especially, *δ*-TOS showed lower apoptogenic activity compared to that of *α*-TOS, where all available positions on the aromatic ring are substituted with methyl groups, although the protein kinase C inhibitory activity of the *α*-tocopheryl-containing analogues also contributes to the superior proapoptotic activity of *α*-TOS within this group of agents (Neuzil *et al*, 2001c).

For VE analogues with saturated aliphatic side chains, the presence of a chargeable ester group, such as succinyl or maleyl, appears essential for apoptosis induction. However, polyunsaturated forms of VE may exert proapoptotic activity even with the redox-active hydroxyl group present. This is the case of *γ*-T3H, which, unlike *α*-T3H, causes apoptosis in a variety of malignant cells (Yu *et al*, 1999; this report). An even stronger proapoptotic effect was observed for *γ*-T2H, a homologue of *γ*-T3H with the phenyl chain shorter by one isoprenyl unit. The mechanism underlying apoptosis induction by *γ*-T3H has not been studied, but appears to differ from that involved in the action of *α*-TOS. One possibility is that *γ*-T3H acts via inhibition of prenylation (Theriault *et al*, 2002). Indeed, other compounds, which block prenylation, have been shown to be strong apoptogens (Miquel *et al*, 1998; Rioja *et al*, 2000). It is tempting to hypothesise that compounds like *γ*-T3H or *γ*-T2H may prevent carcinogenesis by suppressing prenylation of crucial oncogenes, such as Ras (Adjei 2001), in which case these agents would have prophylactic effects.

One important difference between compounds like *α*-TOS and *γ*-T3H is that while *α*-TOS is largely selective for malignant cells (Neuzil *et al*, 2001b, c; Weber *et al*, 2002), *γ*-T3H is highly toxic towards normal cells, such as primary fibroblasts or cardiomyocytes (JN, unpublished). However, succinylation of *γ*-T3H renders the agent even more apoptogenic, and it will be interesting to determine whether such modification makes the agent selective for malignant cells, as might be expected in analogy to *α*-TOS. If so, this would suggest that the addition of a chargeable ester group on the aromatic ring may be a way of conferring selectivity to these compounds. It is plausible that cultured cancer cells, because of their very high metabolic activity, form a pH gradient across the plasma membrane, promoting higher level of *α*-TOS uptake *in vitro*. These notions are compatible with the concept that inducers of apoptosis, which are weak acids, may be selectively taken up by malignant cells due to the acidic interstitium of the tumour (Gerweck and Seetharam, 1996; Kozin *et al*, 2001).

Overall, our findings indicate that modifications in all three functional domains of VE analogues (cf Figure 1) modulate the proapoptotic efficacy of the agents. Before testing the novel compounds *in vivo*, we need to obtain information about their stability. By analogy to previous results with *α*-tocopheryl succinate (Weber *et al*, 2002), we expect gradual hydrolysis of the esters primarily by hepatic esterases. Our recent data for *α*-TOS suggest that, depending on the dosing regimen, the anticancer form of the drug is retained in the circulation long enough to suppress tumour growth. In any case, data presented in this communication further strengthen the idea that this class of compounds may hold substantial promise for the eventual development of selective and nontoxic antineoplastic drugs, and corresponding experiments are underway.
